# Chronic Alcohol-Induced microRNA-155 Contributes to Neuroinflammation in a TLR4-Dependent Manner in Mice

**DOI:** 10.1371/journal.pone.0070945

**Published:** 2013-08-09

**Authors:** Dora Lippai, Shashi Bala, Timea Csak, Evelyn A. Kurt-Jones, Gyongyi Szabo

**Affiliations:** Department of Medicine, University of Massachusetts Medical School, Worcester, Massachusetts, United States of America; University of Pittsburgh, United States of America

## Abstract

**Introduction:**

Alcohol-induced neuroinflammation is mediated by pro-inflammatory cytokines and chemokines including tumor necrosis factor-α (TNFα), monocyte chemotactic protein-1 (MCP1) and interleukin-1-beta (IL-1β). Toll-like receptor-4 (TLR4) pathway induced nuclear factor-κB (NF-κB) activation is involved in the pathogenesis of alcohol-induced neuroinflammation. Inflammation is a highly regulated process. Recent studies suggest that microRNAs (miRNAs) play crucial role in fine tuning gene expression and miR-155 is a major regulator of inflammation in immune cells after TLR stimulation.

**Aim:**

To evaluate the role of miR-155 in the pathogenesis of alcohol-induced neuroinflammation.

**Methods:**

Wild type (WT), miR-155- and TLR4-knockout (KO) mice received 5% ethanol-containing or isocaloric control diet for 5 weeks. Microglia markers were measured by q-RTPCR; inflammasome activation was measured by enzyme activity; TNFα, MCP1, IL-1β mRNA and protein were measured by q-RTPCR and ELISA; phospho-p65 protein and NF-κB were measured by Western-blotting and EMSA; miRNAs were measured by q-PCR in the cerebellum. MiR-155 was measured in immortalized and primary mouse microglia after lipopolysaccharide and ethanol stimulation.

**Results:**

Chronic ethanol feeding up-regulated miR-155 and miR-132 expression in mouse cerebellum. Deficiency in miR-155 protected mice from alcohol-induced increase in inflammatory cytokines; TNFα, MCP1 protein and TNFα, MCP1, pro-IL-1β and pro-caspase-1 mRNA levels were reduced in miR-155 KO alcohol-fed mice. NF-κB was activated in WT but not in miR-155 KO alcohol-fed mice. However increases in cerebellar caspase-1 activity and IL-1β levels were similar in alcohol-fed miR-155-KO and WT mice. Alcohol-fed TLR4-KO mice were protected from the induction of miR-155. NF-κB activation measured by phosphorylation of p65 and neuroinflammation were reduced in alcohol-fed TLR4-KO compared to control mice. TLR4 stimulation with lipopolysaccharide in primary or immortalized mouse microglia resulted in increased miR-155.

**Conclusion:**

Chronic alcohol induces miR-155 in the cerebellum in a TLR4-dependent manner. Alcohol-induced miR-155 regulates TNFα and MCP1 expression but not caspase-dependent IL-1β increase in neuroinflammation.

## Introduction

According to the WHO the harmful effects of alcohol are major public health concerns across the world [1]. The effects of alcohol on the brain include neuroinflammatory and neurodegenerative changes mediated partially via innate immune responses [2], [3]. Recently microRNAs (miRNAs) have been implicated in the pathogenesis of predominantly neurodegenerative or neuroinflammatory diseases, such as Alzheimer’s or neuroviral infections [4], [5]. MiRNAs are evolutionally conserved, small non-coding RNAs which are involved in various biological processes such as development, differentiation, innate and adaptive immune responses [6]. Mature miRNAs regulate posttranscriptional gene expression mainly via repressing translation or inducing mRNA degradation [7]. Recently other mechanisms, such as posttranslational stabilization of mRNA enabling increased translation, have also been proposed, however the exact mechanism is not fully understood [8].

MiR-155 (miR-155) plays an important role in inflammatory conditions and malignant cell growth [9] and is upregulated in the brain in multiple sclerosis and a cerebral ischemia model [5], [10]. Many miR-155 targets are pro-apoptotic and anti- or pro-inflammatory, and miR-155 expression leads to cell survival and modification of inflammation [9]. At present, there is an ongoing debate whether miR-155 plays a pro- or anti-inflammatory role, but the studies agree that miR-155 does play an important regulatory role in inflammation. Among many anti-inflammatory proteins, miR-155 targets phosphatidylinositol-3,4,4-triphosphate 5 phosphatase-1 (SHIP1) (a negative regulator of TNFα) and suppressor of cytokine signaling-1 (SOCS1) (a negative regulator of cytokines), which subsequently leads to increased inflammatory responses [11]. Furthermore, miR-155 is induced in macrophages, dendritic cells, B- and T-cells after Toll-like receptor (TLR) stimulation [9], [12]. A recent report has shown miR-155 induction upon lipopolysaccharide stimulation in a microglia cell line [6]. However there is evidence that the effect of miR-155 is not solely pro-inflammatory, in dendritic cells miR-155 silencing resulted in increased IL-1β production [12]. Pro-inflammatory targets of miR-155 include myeloid differentiation primary response gene (88) (MyD88) and transforming growth factor beta-activated protein kinase-1 binding protein-2 (TAB2) [13], which are upstream of nuclear factor-κB (NF-κB), their inhibition by miR-155 can lead to decreased NF-κB activation [14]. Conversely, in vitro NF-κB inhibition could prevent alcohol-induced upregulation of miR-155 in Kupffer cells [8].

NF-κB activation has recently been proven to be involved in the pathogenesis of alcohol-induced neuroinflammation [2]. The transcriptional activity of the most abundant form of NF-κB heterodimer, p50/p65, is increased by phosphorylation of its p65 subunit [15]. NF-κB is known to induce the transcription of pro-inflammatory cytokines and chemokines, like tumor necrosis factor-α (TNFα), monocyte chemotactic protein-1 (MCP1) and interleukin-1-beta (IL-1β) [16], [17], all of which are increased in alcohol-induced neuroinflammation [2], [3], [18]. Posttranslational cleavage of pro-IL-1β to mature IL-1β is required for its functional activity and is executed by the inflammasome via caspase-1 activation [19]. TLR activation via danger or pathogen associated molecular patterns (DAMPs and PAMPs) leads to NF-κB activation and consequently increased cytokine production [16]. TLR4 is one of the major pathways involved in alcohol-induced neuroinflammation [3], [18].

The aim of our study was to examine the role of miR-155 in the pathogenesis of alcohol-induced neuroinflammation in vivo. Our novel results suggest that chronic alcohol consumption induces miR-155 in the cerebellum in a TLR4-dependent manner. Furthermore, alcohol-induced TNFα and MCP1 production is miR-155-dependent.

## Materials and Methods

### Animals

This study was approved and conducted according to the regulations of the Institutional Animal Care and Use Committee (IACUC) of the University of Massachusetts Medical School (Worcester, MA). Six to eight weeks old female C57/BL6J wild type (WT); miR-155 knock-out (KO) and toll-like receptor-4 (TLR4) KO mice (backcrossed on a C57/BL6J background) were used. For 5 weeks the animals received 5% (v/v) ethanol (36% ethanol-derived calories) containing Lieber-DeCarli diet (EtOH) or pair-fed diet (PF) with an equal amount of calories where the alcohol-derived calories were substituted with dextran-maltose (Bio-Serv, Frenchtown, NJ) [20]. The daily consumption of the diet was the same in the WT and all KO mouse strains, approximately 10 ml/animal.

### Sample Collection

Blood was collected and animals were sacrificed by cervical dislocation. Cerebella and cerebra were immediately isolated and were snap frozen or stored in RNAlater (Qiagen GmbH, Maryland, USA) for protein or messenger ribonucleic acid (mRNA) and miRNA evaluation, respectively. Serum and brain samples were stored at −80°C.

### Cells

Primary microglia of brain of adult WT mice was isolated similar to Frank et al. [21]. Briefly, after cheek bleeding whole brain was washed in ice-cold PBS containing 2% FBS and 0.2% glucose, minced in Petri dish and homogenized in Tenbroeck homogenizer (Wheaton Industries, Millville, NJ). Homogenate was filtered through a 40 µm cell strainer (BD Biosciences, Bedford, MA) into a 50 ml conical tube and was centrifuged at 1250 RPMI for 5 min at room temperature (RT). Supernatant was discarded and pellet was resuspended in 3 ml 70% Percoll and transferred to a 15 ml conical tube. 6 ml 50% Percoll followed by 2 ml 2% fetal bovine serum (FBS) and 0.2% glucose containing phosphate-buffered saline (PBS) were layered on top of the 70% Percoll cell-suspension and centrifuged at 2400 RPMI for 30 min at RT. The layer containing enriched microglia was collected from the interface between the 70 and 50% Percoll phases and washed twice with 1 ml 2% FBS and 0.2% glucose containing PBS and centrifuged at 1250 RPMI for 5 min at RT. Prior to plating, microglia from two mice were pooled together. Isolated brain microglia were suspended in RPMI containing 10% FBS and plated in 96-well plates at a density of 10^5^ cells/100 µl/well. Non-adherent cells were removed by washing cells with PBS one hour after plating. The purity of microglia was evaluated by FACS analysis, 91.2% of the cells were positive for CD11b staining (data not shown). An immortalized mouse microglia cell-line, generated from WT animals, was also employed [22]. The microglia cell line was plated on 6-well plates at a density of 1×10^6^ cells/1 ml/well. The cell experiments were executed a minimum of two times at least in triplicates.

### In vitro Immune Stimulation

Cells were incubated with media alone or media containing 50 mM ethanol (EtOH) and/or 100 ng/ml lipopolysaccharide (LPS) (Sigma, St. Louis, MO) at 37°C, 5% CO_2_ and harvested 1, 6 or 18 hours after stimulation. Samples were run in triplicates for each condition. At the end of each incubation, media was collected, centrifuged at 1250 RPMI for 5 min at 4°C to remove floating cells and supernatants were stored at −80°C. After washing cells twice with PBS, nuclear and cytoplasmic extracts were isolated or cells were lysed in QIAzol Lysis reagent (Qiagen, Maryland, USA) at −80°C for further mRNA and miRNA extraction.

### Polymerase Chain Reaction (PCR)

RNA was extracted using RNeasy kit (Qiagen, Maryland, USA). cDNA was transcribed from 1 µg of total RNA using Reverse Transcription System (Promega Corp., Madison, WI) in a final volume of 30 µl. SYBR-Green-based real-time quantitative PCR was performed using the iCycler (Bio-Rad Laboratories Inc., Hercules, CA). Comparative threshold cycle (Ct) method was used to calculate expressions relative to WT control groups. The final results were expressed as fold changes between the sample and the controls corrected with internal control, 18S [23]. Primers used for the experiments are listed in Table 1.

**Table 1:** Real-Time PCR Primers.

Target gene	Forward primer (5′>3′)	Reverse primer (5′>3′)
**18S**	GTA ACC CGT TGA ACC CCA TT	CCA TCC AAT CGG TAG TAG CG
**CD68**	CCC ACA GGC AGC ACA GTG GAC	TCC ACA GCA GAA GCT TTG GCC C
**Iba1**	CCG AGG AGA CGT TCA GCT AC	GAC ATC CAC CTC CAA TCA GG
**MCP1**	CAG GTC CCT GTC ATG CTT CT	TCT GGA CCC ATT CCT TCT TG
**Pro-IL-1β**	TCT TTG AAG TTG ACG GAC CC	TGA GTG ATA CTG CCT GCC TG
**TNFα**	CAC CAC CAT CAA GGA CTC AA	AGG CAA CCT GAC CAC TCT CC

The following forward and reverse sequences of primers were used in real-time PCR. CD68: cluster of differentiation 68; Iba1: ionized calcium binding adaptor molecule-1; MCP1: monocyte chemoattractant protein 1; pro-IL-1β: pro-interleukin-1β; TNFα: tumor necrosis factor-α.

### MiRNA Analysis

Tissue samples were lysed in QIAzol Lysis reagent (Qiagen, Maryland, USA), homogenized with stainless steel beads (Qiagen, Maryland, USA) in TissueLyser II (Qiagen, Maryland, USA) and incubated on ice for five minutes followed by miRNA isolation using Direct-zol RNA MiniPrep kit with on column DNA digestion (Zymo Research Corp., California, USA). Reverse transcription (30 min - 16°C; 30 min - 42°C; 5 min - 85°C) was performed in Eppendorf Realplex Mastercycler (Eppendorf, New York, USA) using 10 ng RNA, TaqMan primers and MiRNA Reverse Transcription Kit followed by quantitative RT-PCR (10 min −95°C; 40 cycles of 15 sec −95°C; 1 min −60°C) in iCycler (Bio-Rad Laboratories) using TaqMan Universal PCR Master Mix and mouse primers for snoRNA202 as normalizing control, miR-125b, miR-132, miR-146a and miR-155. Relative expression was calculated by Ct method.

### Enzyme-linked Immunosorbent Assay (ELISA)

Tissue lysates were prepared from cerebella in RIPA buffer containing protease and phosphatase inhibitors (1 mM PMSF, 1 mM NaF, 2 mM Na3VO4, 20 mM Na_4_P_2_O_7_ (Sigma-Aldrich, St.Louis, MO), protease and phosphatase inhibitor tablet (Roche Diagnostics, Indianapolis, IN)). First, the tissue was homogenized with stainless steel beads (Qiagen, Maryland, USA) in TissueLyser II (Qiagen, Maryland, USA) then clarified by centrifugation. The tissue lysate supernatant was stored at −80°C. Protein level was measured by ELISA reader using Bio-Rad protein assay dye reagent concentrate (Bio-Rad Laboratories Inc., Hercules, CA). TNFα (BD Biosciences, San Diego, CA), MCP1 (BioLegend Inc., San Diego, CA) and IL-1β (R&D Systems, Inc., Minneapolis, MN) were measured in whole tissue lysates.

### Enzyme-activity Assay

Caspase-1 colorimetric assay was used to determine the enzymatic activity (R&D Systems, Inc., Minneapolis, MN) from cerebellar tissue lysates.

### Western-blot

Tissue lysates were run on 12.5% polyacrylamide gel. Proteins were transferred to nitrocellulose membrane overnight then blocked for two hours in blocking buffer-1 or 2. Primary antibodies against IL-1β (R&D Systems, Inc., Minneapolis, MN), p65, phospho-p65 (Cell Signaling Technology, Inc. Danvers, MA) and beta-actin (Abcam, Cambridge, MA) were used overnight at 4°C at different dilutions varying from 1∶100 to 1∶10,000 in blocking buffer-1 or 2, followed by three washing steps. For detection, appropriate goat anti-rat, anti-rabbit or anti-mouse secondary HRP-linked antibodies (Santa Cruz Biotechnology Inc., Santa Cruz, CA) were used for one hour at a dilution rate of 1∶5000 in blocking buffer-1 or 2. The immunoreactive bands were detected by chemiluminescence using Pierce ECL Western blotting substrate (Pierce Biotechnology, Rockford, IL) and LAS-4000IR Ver.2.02 (Fujifilm Corp., USA). The results were quantified by densitometric analysis using Multi Gauge Ver.3.2 image software (Fujifilm Corp., USA). Blocking buffer-1: 0.1% TWEEN-20 TBS 5% milk. Blocking buffer-2: 0.1% TWEEN-20 TBS 5% BSA.

### Electromobility Shift Assay (EMSA)

End labeling of double-stranded NF-κB oligonucleotide, 5′AGTTGAGGGGACTTTCGC3′ was accomplished by treatment with T4 polynucleotide kinase in the presence of γ^32^P-ATP (PerkinElmer, Waltham, MA), followed by purification on a polyacrylamide copolymer column (Bio-Rad). Microglial nuclear extract (2.5 µg) or cerebellar whole cell lysate (5 µg) was incubated with 1 µl labeled oligonucleotide (50,000 cpm) and 4 µl dI-dC (Affymetrix Inc., Santa Clara, CA) and 5X gel buffer (containing 20 mM HEPES pH 7.9 (Sigma, St. Louis, MO), 50 mM KCl (Sigma, St. Louis, MO), 0.1 mM EDTA (Boston BioProducts Inc., Ashland, MA), 1 mM DTT (Sigma, St. Louis, MO), 5% glycerol (Fisher Scientific, Fair Lawn, NJ), 200 µg/ml BSA in sterile water), a 20 µl final volume was reached by adding nuclease-free water. For supershift analysis, 2 µg of anti-p65 antibody (SantaCruz Santa Cruz Biotechnology Inc., Santa Cruz, CA) was included in the binding reaction 30 minutes prior to labeling. For cold competition reaction a 20-fold excess of specific unlabeled double-stranded probe was added to the reaction mixture 20 minutes prior to adding the labeled oligonucleotide. Samples were incubated at room temperature for 20 minutes. Reactions were run on a 4% polyacrylamide gel. Gels were then dried and exposed to an X-ray film at −80°C for 6 hours or overnight where appropriate. Kodak X-OMAT 2000A Processor was used for film development in the darkroom. The films were scanned and densitometry was performed on the images using Multi Gauge Ver.3.2 image software (Fujifilm Corp., USA) [24].

### Statistical Analysis

Since the data was not normally distributed, statistical analysis was performed using Kruskal-Wallis nonparametric test. Data are shown as average ± standard error of the mean (SEM) and differences were considered statistically significant at *p*≤0.05. The experiments were performed a minimum of two times.

## Results

### Pro-inflammatory Cytokines and microRNAs are induced in Alcohol-fed Mice in the Cerebellum

**Figure 1:**
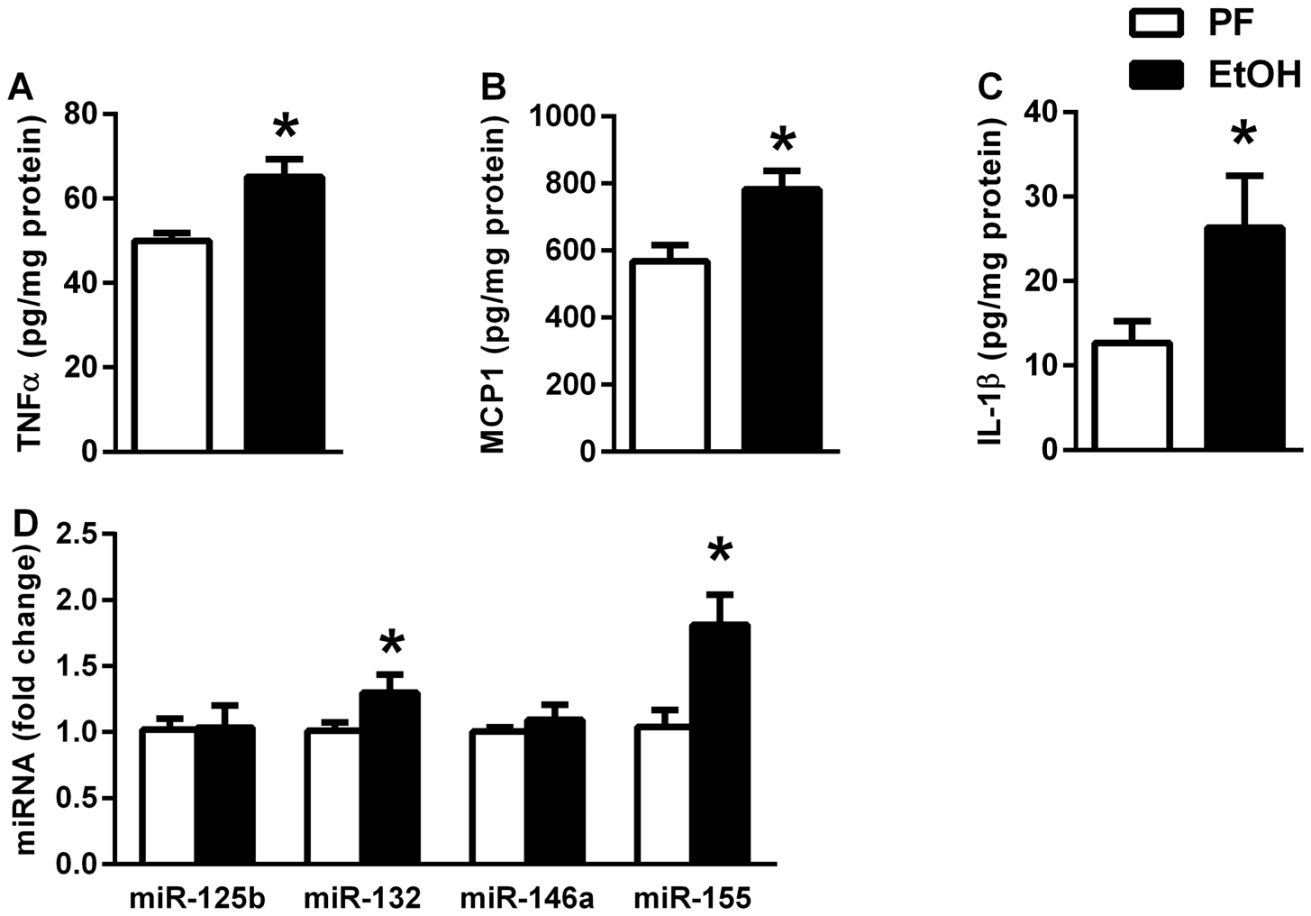
Pro-inflammatory cytokines and microRNAs are increased in alcohol-induced brain injury. WT mice were fed with control (PF, n = 7) or EtOH (n = 8) diet for 5 weeks. Inflammatory cytokines, TNFα (A), MCP1 (B) and IL-1β (C) were measured by specific ELISAs on whole cerebellar lysates, and corrected with total protein. Various microRNAs (125b, 132, 146a, 155) were measured by real-time PCR on whole cerebellar miRNA extract and corrected with snoRNA202 (D). Bars represent mean±SEM (*: *p* value<0.05 relative to appropriate PF controls by Kruskal-Wallis non-parametric test).

Previous reports have shown that neuroinflammation is present and proinflammatory cytokines are upregulated in chronic alcoholic brains in mice as well as humans [2], [3], [18]. We found significant induction of TNFα, MCP1 and IL-1β protein in chronic alcohol feeding compared to control mice in the cerebellum (Figure 1A–C). MicroRNAs (miRNAs) are small non-coding RNAs with regulatory function including modulation of inflammation and cytokine production [25]. MiR-125b, -132, -146a and -155 have been shown to be altered in the LPS-induced inflammatory pathway [25], [26]. We found a significant increase in miR-155 and miR-132, but no change in miR-125b or miR-146a in chronic alcohol feeding compared to control mice in the cerebellum (Figure 1D).

### MicroRNA-155 Deficiency Protected Mice from Ethanol-induced Proinflammatory Cytokine Increase in the Cerebellum

**Figure 2:**
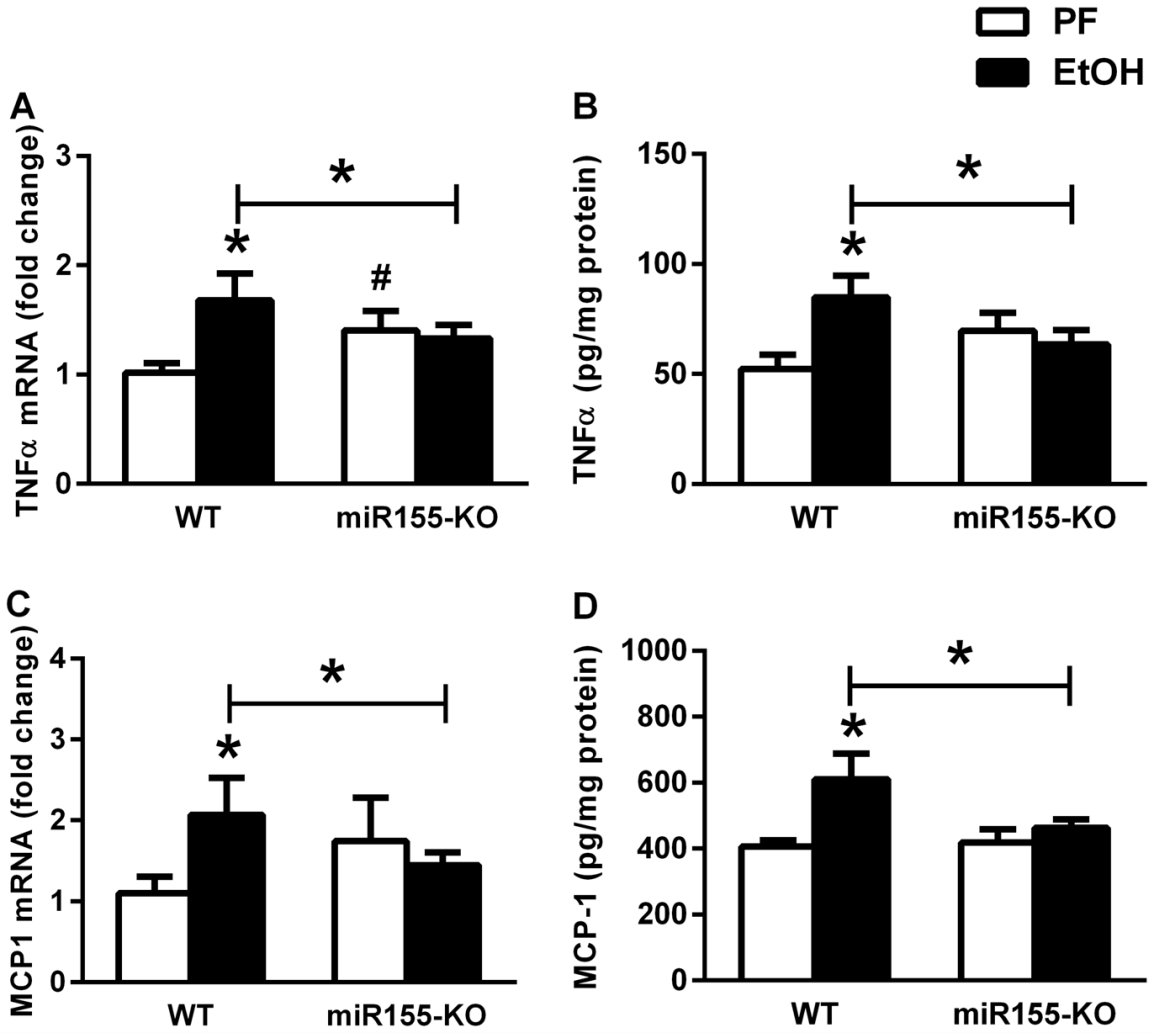
MicroRNA-155 deficiency protects from alcohol-induced TNFα and MCP1 in mouse cerebellum. WT (n = 6 or 7) or miR-155-KO (n = 5 or 10) mice were fed with control (PF) or EtOH diet for 5 weeks, respectively. Pro-inflammatory cytokines, TNFα (A) and MCP1 (C) mRNAs were assessed by real-time PCR from whole cerebellar RNA extract and corrected with 18S. TNFα (B) and MCP1 (D) proteins of whole cerebellar lysates were measured by specific ELISAs and corrected with total protein. Bars represent mean±SEM (*, #: *p* value<0.05 relative to appropriate PF or WT controls, respectively, by Kruskal-Wallis non-parametric test).

MiR-155 can increase TNFα mRNA half-life in RAW macrophage cell line contributing to inflammation [8]. To evaluate the effect of miR-155 on alcohol-induced neuroinflammation, we employed miR-155 deficient mice. In contrast to WT mice, alcohol-fed miR-155-KO mice showed no increase in TNFα and MCP1 both at mRNA and protein levels compared to pair-fed controls (Figure 2A–D).

### Inflammasome Activation and IL-1β Increase is Independent of miR-155 in Alcohol-fed Mouse Cerebellum

**Figure 3:**
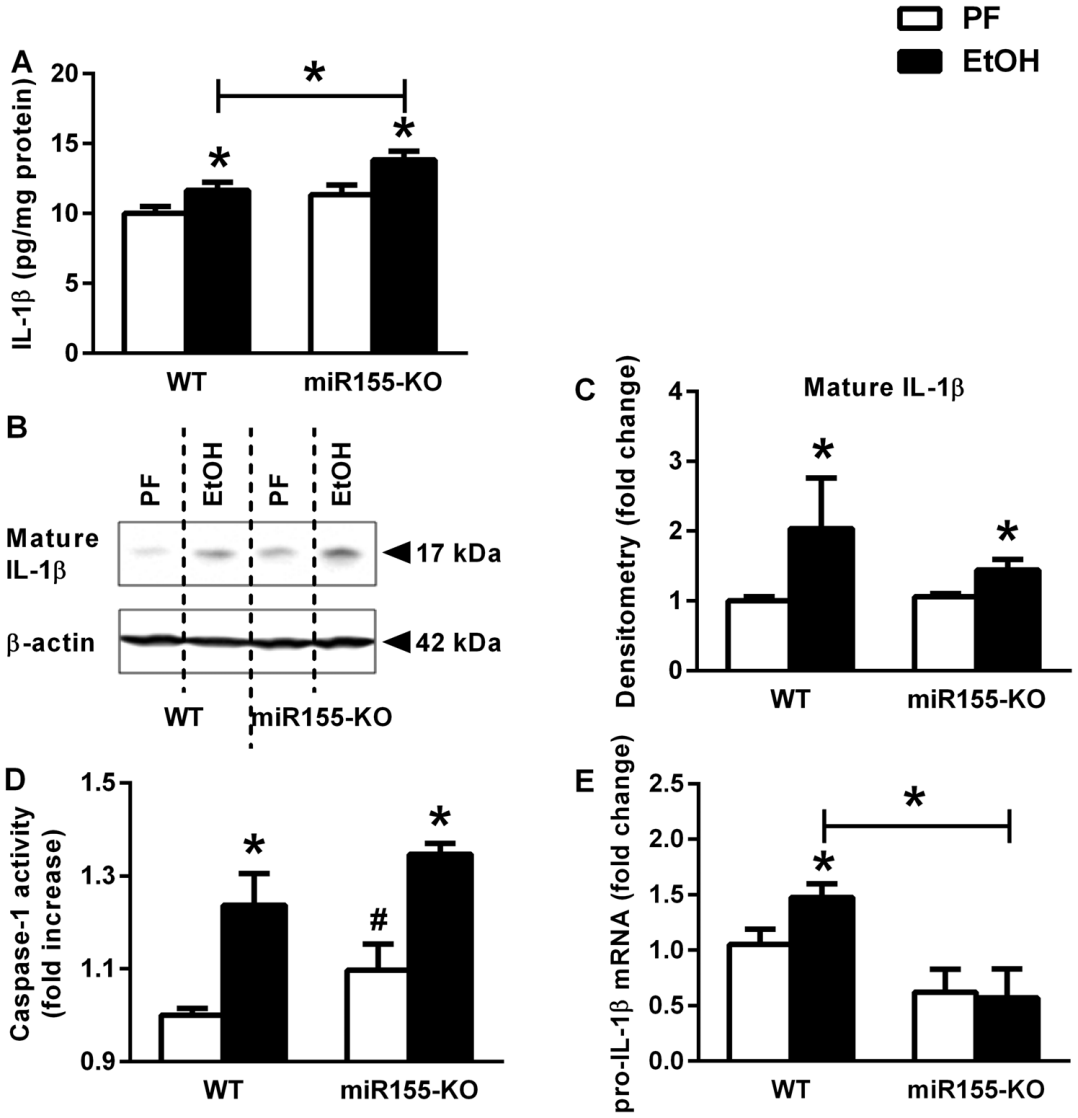
MicroRNA-155 KO mice are not protected from alcohol-induced IL-1β increase in the cerebellum. WT (n = 6 or 7) or miR-155-KO (n = 5 or 10) mice were fed with control (PF) or EtOH diet for 5 weeks, respectively. Inflammatory cytokine, IL-1β was measured by specific ELISA on whole cerebellar lysates and corrected with total protein (A). Mature IL-1β protein of whole cerebellar lysates was assessed by Western blot using β-actin as loading control (B), and further quantified by densitometry (C) which represents six to ten samples per group. The inflammasome activity was measured by caspase-1 colorimetric assay from whole cerebellar lysates and corrected with total protein (D). Pro-IL-1β mRNA was assessed by real-time PCR from whole cerebellar RNA extract, corrected with 18S (E). Bars represent mean±SEM (*, #: *p* value<0.05 relative to appropriate PF or WT controls, respectively, by Kruskal-Wallis non-parametric test).

Recently, we showed inflammasome activation and consequent IL-1β production in the brain of alcohol-fed mice [18]. Interestingly, alcohol-fed miR-155 KO mice showed similar induction of caspase-1 activation and IL-1β protein increase to WT mice (Figure 3A–D), suggesting that caspase-1 and IL-1β are regulated independent of miR-155. However, there was no change in pro-IL-1β mRNA expression in ethanol-fed miR-155-KO compared to control mice (Figure 3E). This observation was similar to the protection from TNFα and MCP1 protein induction but incomplete (partial) protection of caspase-1 activation and IL-1β protein in TLR4-KO mice [18].

### MicroRNA-155 Deficiency Protected Alcohol-fed Mice from NF-κB Activation in the Cerebellum

**Figure 4:**
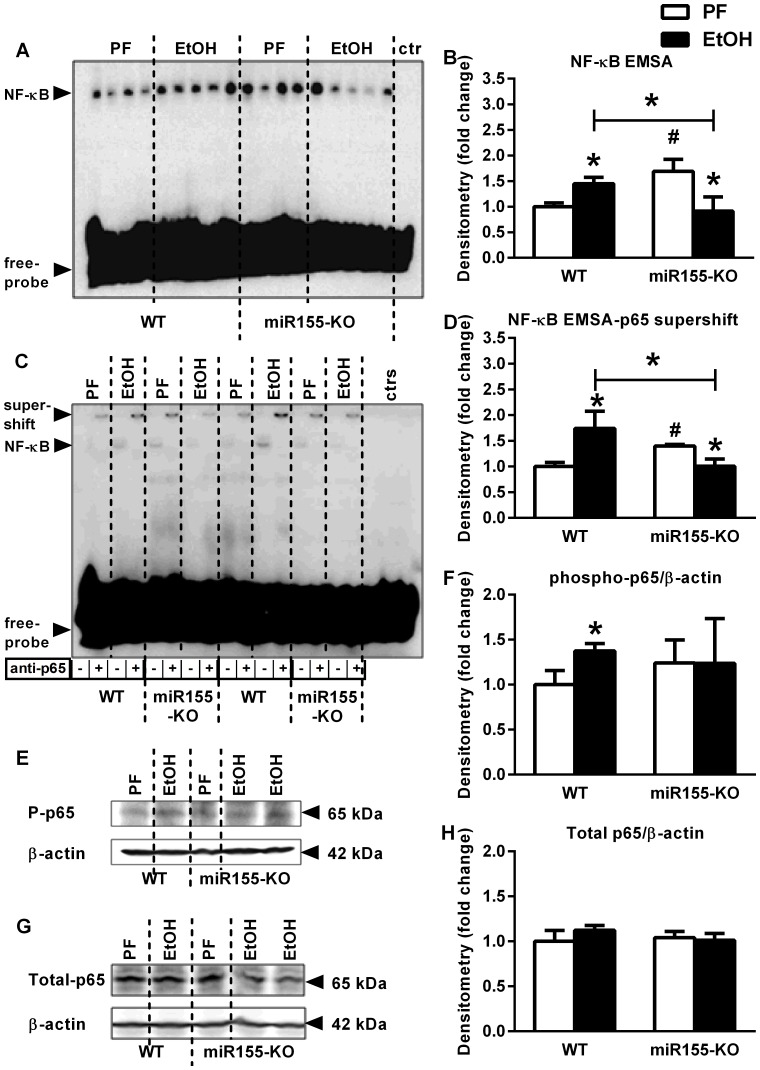
MicroRNA-155 deficiency protects from alcohol-induced NFκB activation in mouse cerebellum. WT (n = 6 or 7) or miR-155-KO (n = 5 or 10) mice were fed with control (PF) or EtOH diet for 5 weeks, respectively. NF-κB activity of whole cerebellar lysates was assessed by EMSA for NF-κB (A–B) and supershift with anti-p65 antibody (C–D), loading equal amounts of protein, using EtOH-fed cerebellar sample for cold competition control (ctr), and further quantified by densitometry. Phosphorylated-p65 (E–F) and total-p65 (G–H) protein of whole cerebellar lysates was assessed by Western blot, using β-actin as loading control, and further quantified by densitometry which represents six to ten samples per group. Bars represent mean±SEM (*, #: *p* value<0.05 relative to appropriate PF or WT controls, respectively, by Kruskal-Wallis non-parametric test).

To evaluate the mechanism by which miR-155 regulates cytokine and chemokine production, we evaluated NF-κB activation. NF-κB is a major regulator in proinflammatory pathways and can upregulate multiple proinflammatory cytokines and chemokines, including TNFα, MCP1 and pro-IL-1β [16], [17]. In addition, NF-κB activation mediates induction of miR-155, in turn miR-155 can decrease NF-κB activity [8], [13]. Activation of NF-κB occurs by phosphorylation of p65, part of the p50/p65 NF-κB heterodimer and its translocation to the nucleus. [15]. We found increased NF-κB DNA binding (measured by EMSA) in the cerebellum of ethanol-fed WT mice compared to controls but no increase in alcohol-fed miR-155-KO mice compared to their PF controls (Figure 4A–B). Furthermore, supershift analysis with p65 antibody showed increased p65 DNA binding in alcohol-fed wild-type but not in miR-155-KO mice compared to pair-fed controls (Figure 4C–D). Consistent with increased NF-κB DNA binding, phosphorylated-p65 levels were also increased in the brains of alcohol-fed WT mice, but there was no increase in the brains from alcohol-fed miR-155-KO mice compared to appropriate controls (Figure 4E–F), while alcohol-feeding did not change total-p65 levels in the brain (Figure 4G–H).

### Induction of miR-155 is TLR4-dependent in Cerebella from Chronic Alcohol-fed Mice

**Figure 5:**
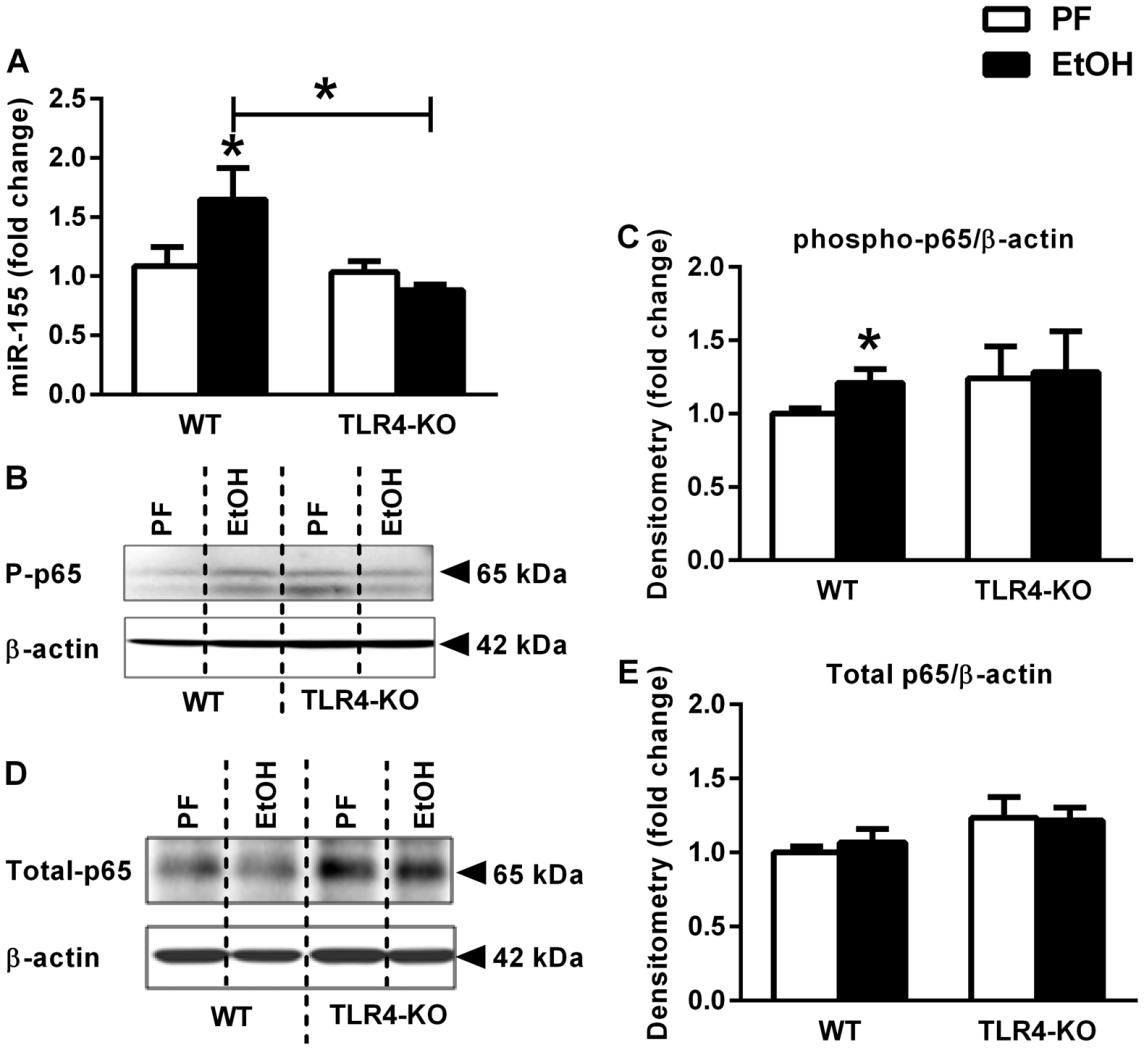
Induction of microRNA-155 is TLR4 dependent in alcohol-fed mouse cerebellum. WT (n = 8 or 7) and TLR4-KO (n = 8 or 13) mice were fed with control (PF) or EtOH diet for 5 weeks, respectively. MiR-155 (A) was assessed by real-time PCR from whole cerebellar miRNA extract, corrected with snoRNA202. Phosphorylated-p65 (B–C) and total-p65 (D–E) protein of whole cerebellar lysates was assessed by Western blot, using β-actin as loading control, and further quantified by densitometry which represents six to twelve samples per group. Bars represent mean±SEM (*: *p* value<0.05 relative to appropriate PF or WT controls, by Kruskal-Wallis non-parametric test).

DAMPs and PAMPs are major inducers of inflammation via receptors, like the Toll-like receptor (TLR) family [27]. Previous reports have shown that TLR4 can activate NF-κB [16] and can also induce miR-155 upregulation [9], [12]. Furthermore alcohol-induced neuroinflammation can be triggered by TLR4 [3]. Here we tested whether TLR4 was required for alcohol-induced upregulation of miR-155. Alcohol-fed TLR4 KO mice had no increase in miR-155 compared to control mice in the cerebellum (Figure 5A). Furthermore, TLR4-KO mice had no NF-κB activation indicated by phosphorylated-p65 compared to control mice in the cerebellum (Figure 5B–C), and there was no change in total-p65 levels (Figure5 D–E). Moreover, recently we showed that alcohol-induced TNFα and MCP1 production was prevented in TLR4-KO mice in the cerebellum [18].

### Induction of miR-155 is TLR4 Dependent in Mouse Microglia

**Figure 6:**
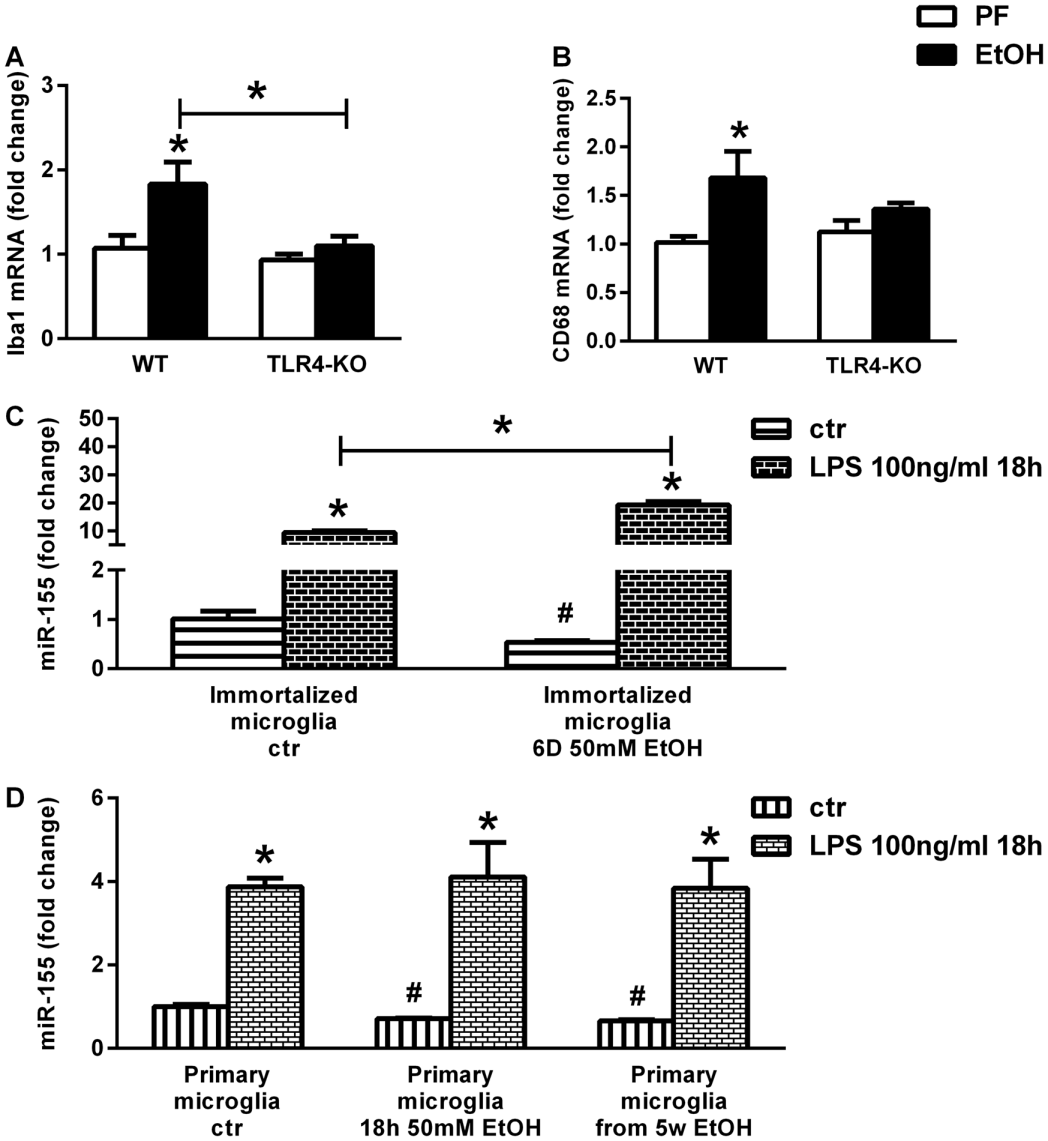
Induction of microRNA-155 is TLR4-dependent in microglia. WT (n = 8 or 7) and TLR4-KO (n = 8 or 13) mice were fed with control (PF) or EtOH diet for 5 weeks, respectively. Microglia markers, Iba1 (A) and CD-68 (B), were assessed by real-time PCR from whole cerebellar RNA extract, and corrected with 18S. WT mouse immortalized microglia cells incubated with or without 50 mM ethanol for 6 days were stimulated with 100 ng/ml LPS for 18 hours. MiR-155 was assessed by real-time PCR of cellular miRNA extracts, corrected with snoRNA202 (C). Primary microglia cells were isolated from WT (n = 10 or 9) mice, fed with control (PF) or EtOH diet for 5 weeks, respectively. Prior to plating, cells were pooled from two brains. Mouse primary microglia cells were stimulated with 100 ng/ml LPS for 18 hours. Microglia from pair-fed mice was also challenged with 50 mM ethanol in vitro for 18 hours. MiR-155 was assessed by real-time PCR of cellular miRNA extracts, corrected with snoRNA202 (D). Bars represent mean±SEM (*: *p* value<0.05 relative to appropriate PF or WT controls, by Kruskal-Wallis non-parametric test).

In a previous study microglia cell line stimulation with TLR4-ligand, LPS, resulted in increased miR-155 expression [6]. We tested whether TLR4 stimulation could directly induce miR-155 in microglia as we found increased mRNA expression of the microglia markers, CD68 and ionized calcium binding adaptor molecule-1 (Iba1), in chronic ethanol-fed WT mice compared to WT control-fed mice in cerebellum, but no change in TLR4-KO mice (Figure 6A–B). The WT immortalized mouse microglia cell line showed increased miR-155 expression upon stimulation with the TLR4-ligand, lipopolysaccharide (LPS) (Figure 6C). Similar results were found in primary mouse microglia (Figure 6D). Ethanol alone decreased miR-155 levels (Figure 6C–D). However ethanol treatment resulted in greater fold induction of miR-155 by LPS in immortalized microglia (from 9.4 to 38.6) and in primary microglia (from 3.8 to 5.85) when compared to cells in media alone, suggesting that alcohol augments LPS-induced miR-155 induction.

## Discussion

Chronic ethanol feeding results in neuroinflammatory changes in cortical, hippocampal and cerebellar brain regions [2], [3], [18]. Increasing evidence suggests that long-term neurodegenerative changes in the cerebellum of alcoholics are not solely due to lack of dietary factors [18], [28]. The neuroinflammatory changes include induction of pro-inflammatory cytokines and chemokines, DAMPs, NF-κB, inflammasome and inducible nitric oxide synthase (iNOS) activation, nictoinamide adenine dinucleotide phosphate (NADPH)-oxidase and reactive oxygen species mediated pathways [2], [3], [18]. The role of miRNAs in the pathogenesis of neurological diseases is gaining increased attention [4], [5].

MiRNAs are involved in the modulation of innate and adaptive immune responses and regulate inflammatory pathways [6]. MiR-155 and miR-132 have broad pro-inflammatory effects, while miR-125b and miR-146a are negative regulators of inflammation in most cell types [29], [30]. Here we found miR-132 and miR-155 upregulation and no changes in miR-125b or miR-146a levels in the cerebellum after chronic alcohol feeding suggesting that miR-132 and miR-155 are involved in the pathophysiology of alcohol-induced neuroinflammation. We show for the first time that miR-155-KO mice were protected from alcohol-induced TNFα and MCP1 induction in the cerebellum. This is consistent with other reports where silencing of miR-155 in LPS treated microglia cell line resulted in decreased TNFα induction, whereas IL-1β levels remained unaffected [6]. Consistent with our findings, miR-155 over-expressing mice showed increased TNFα production upon LPS challenge [30]. MiR-155-KO mice have been reported to have immune deficiencies, including impaired T and B cell development and antigen presentation by dendritic cells. These mice are also prone to developing lung fibrosis and are less resistant to certain bacterial challenges [31], [32]. However, miR-155-KO mice also showed resistance to rheumatoid arthritis and experimental autoimmune encephalomyelitis [33], [34]. These observations together with our results suggest that miR-155 is an important molecular regulator of neuroinflammation induced by alcohol.

A common element in regulating pro-inflammatory gene expression is the activation of NF-κB [16], [17]. NF-κB has binding sites on the promoter regions of IL-1β, TNFα and MCP1 genes [16], [17]. Furthermore, miR-155 is induced by NF-κB activation and we previously reported that in liver resident macrophages, Kupffer cells, miR-155 was induced by chronic alcohol [8]. Moreover, miR-155 induction in Kupffer cells by ethanol or by stimulation with the TLR4 ligand, LPS, was NF-κB dependent [8]. Here we found that in contrast to WT mice, TLR4-KO mice had no induction in miR-155 expression upon alcohol feeding. These observations indicate that the miR-155 regulated pathway is TLR4-dependent and the TLR4-mediated inflammatory pathway is likely miR-155 mediated in our model. Our current data also suggest that miR-155 induction in the brain is TLR4-dependent and involves NF-κB activation. The baseline level of NF-κB activation was somewhat higher in miR-155-KO mice compared to WT, but it did not increase upon alcohol-feeding, and did not affect the baseline protein levels of TNFα, MCP1 or IL-1β supporting the notion for the miR-155-dependent induction by alcohol. Interestingly, in some studies, miR-155 was found to down-regulate NF-κB activation [14].

While miR-155 deficiency protected from alcohol-induced TNFα and MCP1 increase in the brain, it failed to prevent alcohol-induced IL-1β production and caspase-1 activation. Recently we showed that alcohol-fed TLR4-KO mice had similar protection from TNFα and MCP1 protein induction and lack of protection from caspase-1 activation IL-1β protein increases [18]. Unlike TNFα and MCP1, IL-1β increase and caspase-1 activation were not prevented by miR-155 deficiency, which is consistent with reports showing increased IL-1β production in dendritic cells after miR-155 inhibition [12]. Pro-IL-1β mRNA levels were not increased in miR-155-KO mice most likely due to the lack of NF-κB activation. While IL-1β protein production is largely dependent on caspase-1 activation, pro-IL-1β mRNA induction is NF-κB mediated [35]. Inflammasome activation is induced in alcohol-fed mouse brains via PAMPs and DAMPs, like high mobility group box protein 1 (HMGB1) [18]. IL-1β protein level has not been affected by the deletion of either TLR4 or miR-155, which might indicate that the inflammasome mediated pathway has a distinctive regulatory pattern from that of TNFα and MCP1.

Previous reports show activation of microglia and astrocytes along with neuronal changes and cell death in alcoholic brains in both humans and animals [3], [36]. We found upregulation of miR-155 in LPS-stimulated microglia cell line as well as primary microglia isolated from pair-fed or alcohol-fed mice. Consistent with our findings, others have shown that miR-155 is increased upon LPS stimulation in an N9 microglia cell line, reducing its target gene SOCS1 [6]. Ethanol alone decreased miR-155 in microglia, but ethanol treated cells or cells from ethanol-fed animals had higher miR-155 fold-induction by LPS, suggesting a sensitization to PAMPs and potential TLR4-inducing DAMPs. The fact that miR-155 was decreased in primary microglia from alcohol-feeding might be attributable to the 18 hours incubation period with media only, which is devoid any of the DAMPs or PAMPs that would be present in vivo. To address this question, processing of miRNA immediately after microglia isolation would be necessary. Another plausible explanation is that other cells, for example astrocytes, may be involved in miR-155 induction in the brain, but this awaits further investigation.

In summary, we report for the first time that miR-155 is induced in alcohol-fed mice in the brain. The induction of miR-155 in the cerebellum is TLR4-dependent. Furthermore, cerebellar induction of TNFα and MCP1 is miR-155-dependent, however, induction of mature-IL-1β is miR-155 independent in chronic alcohol feeding. We propose that miR-155 silencing might have a therapeutic role in the improvement of alcohol-induced neuroinflammation and further work on this field is warranted.
